# SYNGR3 Accelerates α‐Synuclein Aggregation and Neurodegeneration in Parkinson's Disease

**DOI:** 10.1002/cns.70842

**Published:** 2026-03-24

**Authors:** Xin Wang, Jiaolong Yang, Ziku Wang, Zhichao Liu, Wei Tan, Zhentao Zhang

**Affiliations:** ^1^ Department of Neurology Renmin Hospital of Wuhan University Wuhan China; ^2^ Geriatric Hospital Affiliated to Wuhan University of Science and Technology Wuhan China; ^3^ Taikang Center for Life and Medical Sciences Wuhan University Wuhan China

**Keywords:** α‐synuclein, mitochondrial dysfunction, parkinson's disease, protein aggregation

## Abstract

**Background and Aims:**

The aggregation of α‐synuclein (α‐syn) is a central event in Parkinson's disease (PD) pathogenesis. However, the cellular factors that initiate and accelerate the process are not fully understood. Synaptogyrin‐3 (SYNGR3) is a synaptic vesicle protein whose role in α‐syn pathology remains unexplored. This study investigated whether SYNGR3 is a key factor triggering the pathological process of PD.

**Methods:**

This study investigated the expression of SYNGR3 in the brains of transgenic A53T α‐syn mutant mouse line M83 (TgA53T) PD model mice using Western blot. The direct interaction between SYNGR3 and α‐syn was assessed by GST pull‐down assays. This study examined the effect of SYNGR3 on α‐syn aggregation kinetics and fibril stability in vitro through the thioflavin T (Th T) assays and proteinase K (PK) digestion. By overexpressing or knocking down SYNGR3 in HEK‐293 cells stably transfected with α‐syn, primary neurons, and TgA53T mice, the effects of enhanced or deficient function of SYNGR3 on α‐syn pathology, synaptic integrity, mitochondrial function, and motor behavior were evaluated.

**Results:**

SYNGR3 levels were significantly elevated in an age‐dependent manner in the striatum of TgA53T mice. The study found that SYNGR3 directly interacts with the central region of α‐syn and accelerates its aggregation into fibrils that are more resistant to PK digestion. Overexpression of SYNGR3 exacerbated α‐syn aggregation, synaptic protein loss, mitochondrial dysfunction, and apoptosis in cellular models. In vivo, SYNGR3 intensified α‐syn pathology, dopaminergic neurodegeneration, and PD‐like motor deficits. Conversely, knockdown of SYNGR3 effectively alleviated these pathological and behavioral impairments.

**Conclusion:**

This study identifies SYNGR3 as a novel and critical promoter of α‐syn aggregation and neurotoxicity. These findings establish SYNGR3 as a key contributor to PD pathogenesis and highlight its potential as a therapeutic target for intervention.

## Graphical abstract

This study is the first to demonstrate that SYNGR3 interacts with α‐synuclein and identify SYNGR3 as a key trigger of α‐synuclein aggregation in Parkinson's disease. Targeting SYNGR3 mitigates neurotoxicity and motor deficits, highlighting its promising potential as a therapeutic strategy.

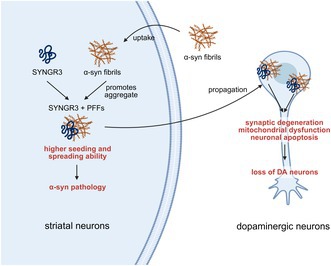

## 1 Introduction

Parkinson's disease (PD) is one of the most common neurodegenerative disorders in elderly individuals [1]. PD is typically characterized by the progressive loss of dopaminergic neurons in the substantia nigra pars compacta (SNpc) and the formation of Lewy bodies (LBs) and Lewy neurites (LNs) [2, 3]. Aggregated α‐synuclein (α‐syn), the major protein component of LBs and LNs, is highly enriched in presynaptic terminals and exists in a soluble state under physiological conditions [4]. Further, α‐syn regulates the stability of the presynaptic membrane, neurotransmitter metabolism, and synaptic vesicle transport [4, 5]. However, in PD, α‐syn undergoes conformational changes that promote pathological aggregation. Extensive evidence indicates that aggregated α‐syn acts as proteinaceous nuclei (‘seeds’) to induce the propagation and cell‐to‐cell transmission of α‐syn pathology [6]. It has been reported that FAM171A2 acts as a key receptor that mediates the uptake of α‐syn pre‐formed fibrils (PFFs) and directly promotes the “seeding” activity and spread of pathological proteins between cells [7]. Despite these insights, the mechanisms that initiate the aberrant aggregation of α‐syn remain elusive. Multiple factors, including genetic mutations, post‐translational modifications and interactions with other proteins, are known to influence both the aggregation of α‐syn [8, 9, 10, 11] and the resulting structural properties of α‐syn fibrils, contributing to disease heterogeneity [12].

Aberrantly aggregated α‐syn species have been reported to disrupt signal transduction in the presynaptic membrane, leading to synaptic dysfunction and neuronal degeneration [13, 14]. This underscores the importance of investigating synaptic proteins that might interact with and modulate α‐syn pathology. Synaptogyrin‐3 (SYNGR3) is a synaptic vesicular membrane protein. It belongs to a multigene family of four integral synaptic vesicle proteins that participate in the maintenance of various functions of synaptic vesicles [15, 16]. Surface‐exposed SYNGR3 binds to various macromolecular substances and mediates their endocytosis and exocytosis [17]. Notably, SYNGR3 has been reported to bind to tau, promoting the formation of insoluble helical proteins and mediating presynaptic dysfunction [18]. Knockdown of SYNGR3 attenuates the synaptic damage caused by tau [19]. In addition, SYNGR3 interacts with the dopamine transporter (DAT), suggesting a potential link to the nigrostriatal pathway vulnerable in PD [20]. However, whether SYNGR3 directly influences α‐syn pathology remains unclear. Given its synaptic location and established role in interacting with aggregation‐prone proteins, we hypothesized that SYNGR3 could be a novel regulator of α‐syn pathology. In this study, we found that the expression of SYNGR3 is increased in PD models. SYNGR3 directly interacts with α‐syn and exacerbates its pathology in vitro and in vivo. The study results uncover a previously unrecognized role for SYNGR3 in driving PD pathogenesis.

## 2 Methods

### 2.1 Mice

Wild‐type (WT) C57BL/6J mice and the transgenic A53T α‐syn mutant mouse line M83 (TgA53T) were obtained from Jackson Laboratory (stock numbers: 0,00,664 and 0,04,479, respectively). Mice were raised in an SPF environment at 22°C. They had free access to food and water and were exposed to a 12‐h light/12‐h dark cycle every day. All procedures were approved by the Institutional Animal Care and Use Committee (IACUC) of Renmin Hospital of Wuhan University.

### 2.2 Purification of α‐Syn and Preparation of Pre‐Formed Fibrils

Recombinant human α‐syn was purified as previously described [21]. His‐tagged α‐syn and SYNGR3 were purified from *Escherichia coli* ( *E. coli* ) through Ni‐NTA affinity chromatography and eluted with approximately 125 mM imidazole. After the purity and concentration of the protein were detected using the SDS‐PAGE and BCA assay, the proteins were incubated at 37°C with shaking at 1000 rpm for 5 days to induce the formation of α‐syn pre‐formed fibrils (PFFs) in the presence or absence of SYNGR3. To prepare α‐syn PFFs, α‐syn monomers were dissolved in phosphate‐buffered saline (PBS) with a final concentration of 1 mg/mL. SYNGR3‐α‐syn PFFs were prepared by adding 0.05 mg/mL or 0.1 mg/mL SYNGR3 into the α‐syn fibrilization reaction. The thioflavin T (Th T) fluorescence assay was performed to monitor the process of fibrilization. In brief, 5 μL samples were diluted with 25 mM Th T and made up to a final volume of 100 μL with PBS and tested at 450 nm excitation and 510 nm emission wavelengths via a SpectraMax plate reader. In this study, all the PFFs used were sourced from the same batch.

### 2.3 Cell Culture and Transfection

HEK293 cells, HEK293 cells stably transfected with GFP‐tagged α‐syn (α‐syn‐HEK293 cells) and SH‐SY5Y cells were cultured in Dulbecco's modified Eagle's medium (DMEM) supplemented with 10% fetal bovine serum and penicillin/streptomycin at 37°C and 5% CO_2_. For transduction, equal final concentrations of α‐syn PFFs were added to Opti‐MEM (Gibco). In parallel, 3 μL of Lipofectamine 2000 (Invitrogen) was mixed with Opti‐MEM, combined with the PFFs containing solution and incubated at room temperature for 15 min. Similarly, His‐tagged SYNGR3 plasmids (GenScript Biotech, Nanjing, China) and control plasmids were transfected into cells. The effects of different treatments on the phosphorylation and aggregation of α‐syn were observed 48 h later by immunofluorescence or immunoblotting.

### 2.4 GST Pull‐Down Assay

Plasmids encoding glutathione‐S‐transferase (GST)‐vector or GST‐tagged full‐length α‐syn or its fragments including GST‐α‐syn‐1‐65, GST‐α‐syn‐66‐140, and GST‐α‐syn‐1‐103 were co‐transfected with His‐tagged SYNGR3 into HEK293 cells. Further, 48 h after transfection, the cells were harvested and lysed. About 40 μl of cell lysates were reserved as input, and the rest of the cell lysates were incubated with glutathione agarose beads overnight at 4°C. The beads were washed five times with 600 μL PBS containing 0.1% Triton X‐100 and then boiled in sodium dodecyl sulfate (SDS) loading buffer for 10 min to elute the bound proteins.

### 2.5 Proteinase K Digestion

Different concentrations of proteinase K (PK) enzyme (0, 2.5, 5, or 10 μg/mL) were separately added to 100 μg of α‐syn PFFs formed with or without SYNGR3 and incubated at 37°C for 30 min. Then, protease inhibitor cocktail (0.1 μg) was added to terminate digestion. These samples were boiled in loading buffer for 10 min. After PK digestion, the bands were detected using the Coomassie blue staining.

### 2.6 Sequential Protein Extraction

Primary neurons and α‐syn‐HEK293 cells were lysed in 1 mL of 1% TritonX‐100 (TX‐100) in Tris‐buffered saline (TBS) (20 mM Tris, 140 mM NaCl, pH 7.4) containing a protease and phosphatase inhibitors cocktail (Sigma, P9599), sonicated and centrifuged at 100,000 × g at 4°C for 1 h. The supernatant was collected as the soluble fraction. Pellets were washed in 1 mL TBS, centrifuged at 100,000 × g at 4°C for 1 h, subsequently resuspended in 0.25 mL of 2% SDS in TBS, and finally sonicated and centrifuged at 100,000 × g at 4°C for 1 h. Mouse brain tissues were lysed in radioimmunoprecipitation assay (RIPA) buffer (Beyotime, P0013B) supplemented with a protease inhibitor cocktail (Sigma Aldrich, P9599) and phosphatase inhibitor (APExBIO, K1015). The homogenates were subsequently centrifuged at 100,000 × g at 4°C for 1.5 h. The pellet was subsequently resuspended in 2% SDS in TBS buffer and centrifuged at 100,000 × g at 4°C for 1 h. Protein levels were normalized using a BCA protein quantitation kit (Thermo Fisher) and analyzed by Western blots using primary antibodies.

### 2.7 RNA Extraction and RT‐PCR

Total RNA was extracted from α‐syn HEK293 cells or primary neurons using the TRIzol reagent (Invitrogen, 15,596,026). The quantity and purity of RNA were determined using a spectrophotometer (NanoDrop Technologies, ND‐1000). Reverse transcription was performed using SuperScript III reverse transcriptase (Thermo Fisher, 18,080,093). The sequences of the gene‐specific primers were referenced from Primerbank and synthesized using Augct Biotechnology. *GAPDH* was used as the internal reference gene. Predenaturation was conducted at 95°C for 5 min, followed by 40 cycles of denaturation at 95°C for 15 s and annealing at 60°C for 15 s. The relative quantification of gene expression was calculated using 2^‐ΔΔCt. Normalization was performed for the control group. The primer sequences used are shown in Table S1.

### 2.8 Immunohistochemistry and Immunofluorescence

Mice were anesthetized and perfused with saline and 4% paraformaldehyde (PFA) for immunostaining. The brain tissues were removed, embedded in paraffin according to different parts, and cut into 4 μm‐thick sections. The sections were deparaffinized in xylene, then hydrated in ethanol with a decreasing gradient and subsequently immersed in antigen retrieval solution (80 mM citric acid and 20 mM sodium citrate, pH 6.0) at 94°C for 20 min to re‐expose the antigen epitopes. The slices were subsequently incubated with 3% hydrogen peroxide for 10 min to eliminate endogenous peroxidase activity. After being blocked with 3% BSA for 30 min, the slices were incubated with primary antibody at 4°C overnight. After rinsing thrice in PBS, the signals in the sections were developed using a DAB staining kit (Absin, abs957). For immunofluorescence staining, a mixture of Alexa Fluor 594‐ and 488‐conjugated secondary antibodies (1:500, Invitrogen, A11005, A11029, 1:500, A31573) was applied. After staining with 4′,6‐diamidino‐2‐phenylindole (DAPI) solution (1:10,000, Biofroxx, Germany, EZ3412B205), images were captured using an Olympus DP80 microscope equipped with TH4‐200 and U‐HGLGPS light sources or a Leica confocal microscope.

### 2.9 Primary Neuronal Culture and Treatment

The dissection and culture of primary neurons were performed according to methods reported previously [22]. In brief, cortical neurons were prepared from E18 wild‐type mouse embryos and cultured in neurobasal medium supplemented with B‐27 (Gibco, 17,504,044), 0.5 mM L‐glutamine (Gibco, A2916801), penicillin, and streptomycin (Gibco, 15,140,122) at 37°C and 5% CO_2_. The neurons were cultured for 7 days in vivo. After the rAAV‐hSyn‐WPRE‐hGH‐pA (AAV‐Vector) or rAAV‐hSyn‐SYNGR3‐2a‐WPRE‐hGH‐pA (AAV‐SYNGR3) was added to the culture medium, the cells were cultured for 4 days. Then, α‐syn PFFs (2.5 μg/mL final concentration) were added to the medium and incubated for 7 days.

### 2.10 Stereotactic Injection

The AAVs encoding SYNGR3 (AAV‐SYNGR3) were prepared by Brain VTA Technology (Wuhan, China). The rAAV‐U6‐shRNA‐SYNGR3‐CMV‐SV40 pA that knocked down SYNGR3 (AAV‐shSYNGR3) was also prepared by Brain VTA Technology. AAV‐SYNGR3, AAV‐vector or AAV‐shSYNGR3 (300 nL) together with 10 μg of sonicated α‐syn PFFs (1 mg/mL) were unilaterally injected into the right striatum of hemizygous TgA53T mice at a speed of 0.3 μL/min with the following coordinates: anteroposterior (AP) = + 0.2 mm, mediolateral (ML) = + 2.0 mm, and dorsoventral (DV) = + 2.8 mm from the bregma. After injection, the needle remained in place for an additional 5 min to ensure diffusion. Mice were then monitored and received postoperative care.

### 2.11 Counting of Dopaminergic Neurons in the Substantia Nigra

The SNpc region of the mouse brain was successively sectioned into 4 μm paraffin slices. Every sixth section was included in the counting procedure for tyrosine hydroxylase (TH)‐positive cells in the SNpc because the volume of each of the six sections was 24 μm thick, which is approximately the diameter of a dopaminergic neuron. Therefore, the total number of cells involved in all the slices is considered the number of dopaminergic neurons in a certain mouse brain. MicroBrightField stereological software (StereoInvestigator v.9.14, MBF Bioscience) was used for cell counts.

### 2.12 Optical Densitometry Analysis

The method for measuring the density of TH‐positive fibers in the striatum was previously reported [23]. Using ImageJ software (version 2.1.0/1.53c), optical density measurements were conducted over the whole range on five coronal layers of the striatum, with equal distances relative to the bregma. The staining signal was calibrated by subtracting cortical baseline levels.

### 2.13 DiI Staining

Dendritic structures were visualized using the 1,1′‐dioctadecyl‐3,3,3′,3′‐tetramethylindocarbocyanine perchlorate (DiI) staining. The neurons were fixed with 4% paraformaldehyde for 15 min and then washed with PBS. DiI powder (Invitrogen, D‐282) was evenly spread on the slides and incubated for 15 min before being washed off with PBS. Finally, samples were observed under a confocal laser microscope (Leica).

### 2.14 Cell Counting Kit‐8 (CCK‐8) Assay

SH‐SY5Y cells were seeded at a density of 1 × 10^4^ cells/100 μL in 96‐well plates. After co‐transfection of α‐syn PFFs with His‐vector or His‐SYNGR3 plasmids and 48 h of incubation, 10 μL of CCK‐8 reagent (Solarbio) was added to each well, and the mixture was incubated at 37°C for 1 h. The optical density (OD 450 nm) values were measured with a SpectraMax plus 384, and the cell viability was expressed as a percentage of control.

### 2.15 Behavioral Tests

To test motor impairments in PD model mice, the mice were subjected to a series of behavioral tests, including the tail suspension test, pole test, rotarod test, grip strength test, and open field test. For the tail suspension test, the mice were monitored by hanging their tails in front of a blank background wall and photographed when they were immobile. The monitoring lasted for up to 2 min. For the pole test, the mice were placed head‐up on a roughened vertical wooden stick with a diameter of 1 cm and a length of 45 cm and allowed to crawl down. The mice were trained for 2 days, and the time from turning to reaching the bottom of the stick was recorded during the final test. In the rotarod test, mice were placed on a rotating rod that rotated uniformly from 5 to 40 rpm within 3 min. The mice were subjected to continuous training for three consecutive days, thrice daily, with at least 30 min of rest between each training session. Finally, the latency to fall was recorded on the test day. For the balance beam test, two beams with flat surfaces were used (length 80 cm and width of 1.6 cm and 0.9 cm). The 1.6 cm wide beam was used for training, with the mice being trained thrice daily for two consecutive days. On the third day, the mice conducted a test on the 0.9 cm wide beam, and the time required to traverse the central 50 cm was recorded.

### 2.16 Data Analysis

Data are presented as means ± SEM from three or more independent experiments and illustrated using GraphPad Prism (version 9.0). Statistical analysis was performed by either Student's *t*‐test (two‐group comparison) or one‐way ANOVA followed by LSD post hoc test (more than two groups). *P* values of < 0.05 were considered as statistically significant.

## 3 Results

### 3.1 The Level of SYNGR3 Is Increased in the Brains of PD Model Mice

**FIGURE 1:**
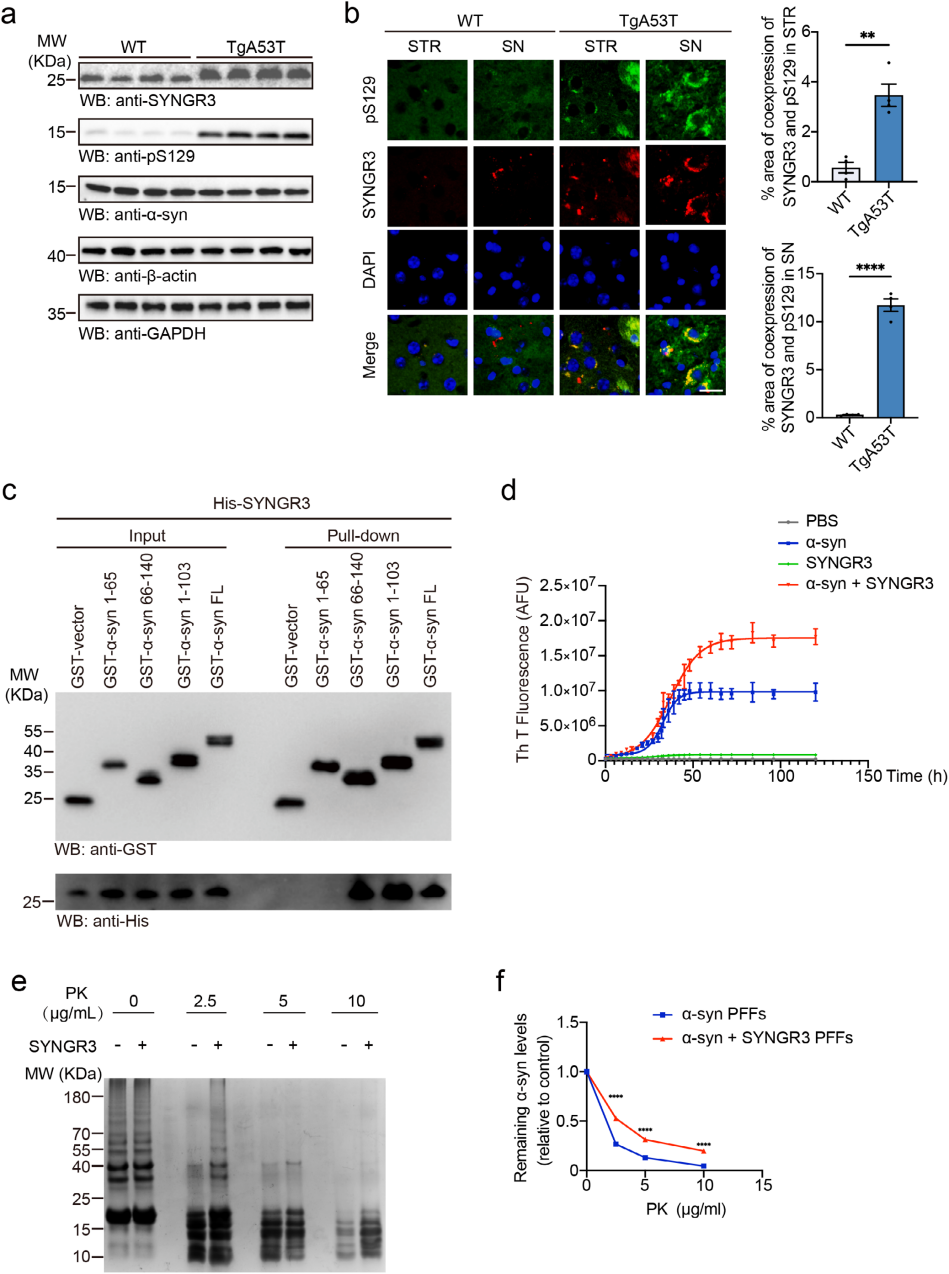
SYNGR3 interacts with α‐syn and promotes its aggregation. a. Representative Western blots showing the levels of SYNGR3 and pS129 in the striatum of 9‐month‐old wild‐type (WT) mice and TgA53T mice. b. Co‐localization of pS129 (green) and SYNGR3 (red) in the striatum (STR) and substantia nigra (SN) of WT and TgA53T mice. Scale bar = 20 μm. The bar graph shows the percentage of area of co‐expression of SYNGR3 and pS129 in STR (*top*) and SN (*bottom*). c. GST pull‐down assay showing the interaction between SYNGR3 and different α‐syn fragments. d. Fibrillization kinetics of 1 mg/mL α‐syn in the presence or absence of 0.1 mg/mL SYNGR3. e. PK digestion patterns of α‐syn PFFs formed in the presence or absence of SYNGR3. Quantification of the remaining α‐syn (*n* = 4 independent experiments). Data are presented as mean ± SEM. *P* values were determined by *t*‐test or one‐way ANOVA followed by Tukey's multiple comparisons test (d). *p < 0.05, **p < 0.01, ***p < 0.001, ****p < 0.0001.

The study first measured the levels of SYNGR3 in the brains of TgA53T mice, which express A53T mutant human α‐syn. Western blot analysis revealed that the levels of SYNGR3 and α‐syn phosphorylated at serine 129 (pS129) were higher in the striatum of 9‐month‐old TgA53T mice than in those of age‐matched control mice (Figure 1a, S1a). Interestingly, SYNGR3 expression increased with age in TgA53T mice but didn't differ significantly across ages in WT mice (Figure S1b). These results indicate a potential role for SYNGR3 in PD pathogenesis.

### 3.2 SYNGR3 Interacts With α‐Syn and Promotes Its Aggregation

Immunofluorescence staining revealed that SYNGR3 co‐localized with phosphorylated α‐syn in the striatum and substantia nigra of TgA53T mice (Figure 1b). To explore the potential interaction between SYNGR3 and α‐syn, the GST‐tagged α‐syn and His‐tagged SYNGR3 plasmids were co‐transfected into HEK293 cells. GST pull‐down assay revealed that SYNGR3 interacts with α‐syn. To map the interaction domain, the GST pull‐down assay using SYNGR3 and different fragments of α‐syn were further performed. The results showed that SYNGR3 bound to the α‐syn (1–103) and α‐syn (66–140) fragments, but not to the α‐syn (1–65) fragment (Figure 1c), suggesting that the central region of α‐syn mediates the interaction.

To investigate whether the interaction between SYNGR3 and α‐syn regulates α‐syn aggregation in vitro, the recombinant α‐syn monomers (1 mg/mL) were incubated in the presence or absence of recombinant SYNGR3 at various concentrations and the aggregation kinetics was monitored using the Th T staining. Notably, SYNGR3 markedly accelerated the aggregation of α‐syn in a concentration‐dependent manner (Figure 1d, S1c). Furthermore, the PK digestion analysis revealed that α‐syn fibrils formed in the presence of SYNGR3 were more resistant to PK than fibrils formed in the absence of SYNGR3 (Figures 1e and 1f). These results indicated that SYNGR3 interacted with α‐syn and promoted its aggregation, forming α‐syn fibrils that were more resistant to protease digestion.

### 3.3 SYNGR3 Facilitates α‐Syn Aggregation and Synaptic Degeneration in Vitro

**FIGURE 2:**
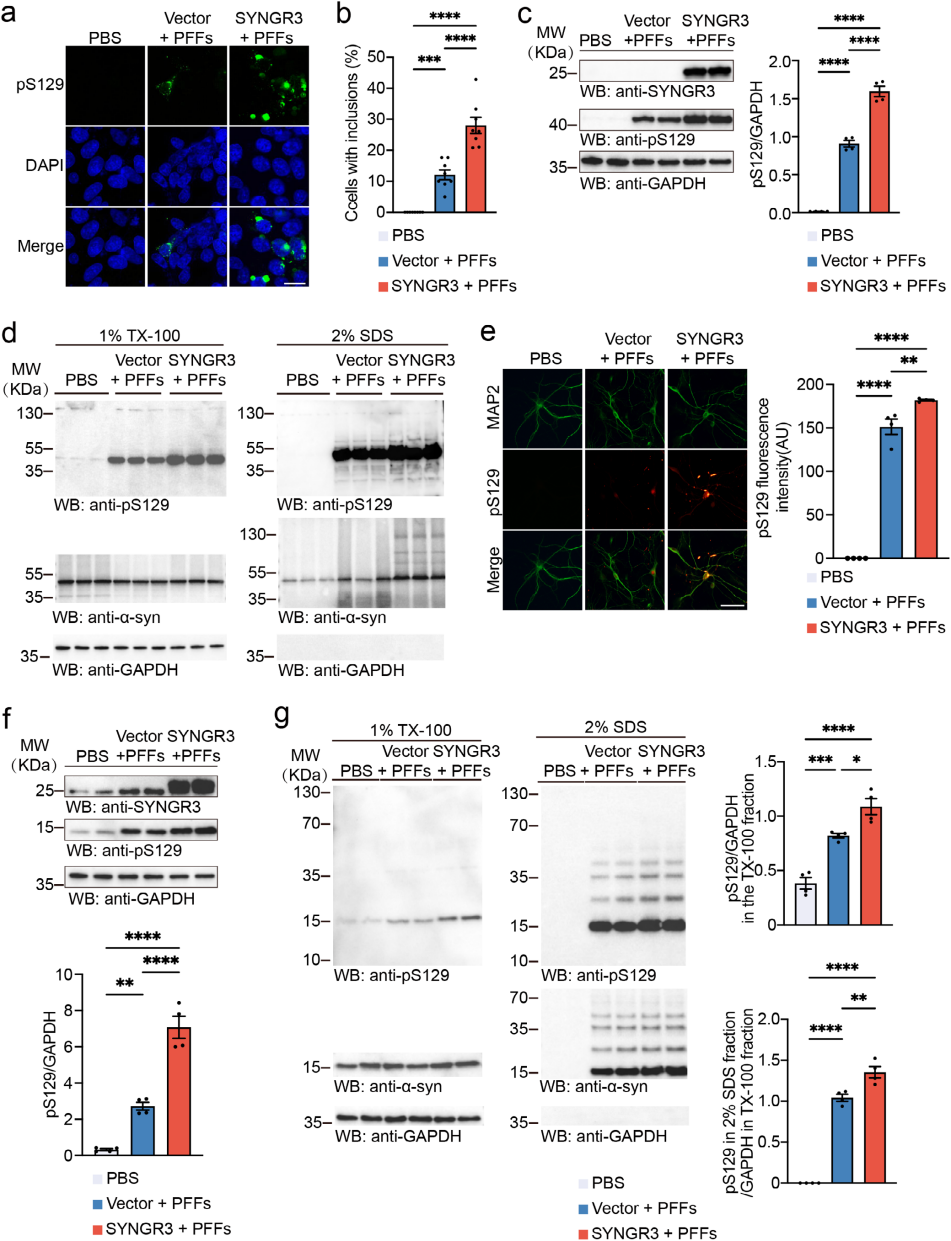
SYNGR3 promotes α‐syn phosphorylation and aggregation. a. Immunofluorescence showing α‐syn aggregates (green) in α‐syn‐HEK293 cells transfected with SYNGR3 plasmids or control plasmids and then treated with PFFs. Scale bar = 20 μm. b. The bar graph shows the quantification of the percentage of cells with inclusions (*n* = 8 independent experiments). c. Western blot analysis of pS129 in α‐syn‐HEK293 cells transfected with His‐SYNGR3 plasmids or control plasmids and then treated with α‐syn PFFs (*n* = 4 independent experiments). d. α‐Syn‐HEK293 cells were transfected with His‐SYNGR3 plasmids or control plasmids and then treated with α‐syn PFFs. The cells were sequentially extracted with 1% TX‐100 and 2% SDS. Western blot analysis shows the levels of pS129 and α‐syn. e. Double‐labeling immunofluorescence of MAP2 (green) and pS129 (red) in primary neurons treated with α‐syn PFFs together with AAV‐SYNGR3 or AAV‐vector. Scale bar = 50 μm. *Right*: Quantification of pS129 fluorescence intensity (*n* = 4 independent experiments). f. Primary neurons were infected with AAV‐vector or AAV‐SYNGR3 and then treated with α‐syn PFFs. Western blot analysis shows pS129 levels in primary neurons. *Bottom*: Quantification of pS129 levels (*n* = 4 independent experiments). g. Primary neurons were sequentially extracted with 1% TX‐100 followed by 2% SDS. Western blot analysis shows the levels of pS129 and α‐syn. *Right*: Quantification of pS129 in each fraction (*n* = 4 independent experiments). Data are presented as mean ± SEM. *P* values were determined by one‐way ANOVA followed by Tukey's multiple comparison test. *p < 0.05, **p < 0.01, ***p < 0.001, ****p < 0.0001.

To determine the effect of SYNGR3 on α‐syn aggregation, the α‐syn‐HEK293 cells used in GFP‐α‐syn form aggregates that can be easily observed under the fluorescence microscopy. The α‐Syn‐HEK293 cells were initially transfected with His‐SYNGR3 plasmids and then exposed to α‐syn PFFs. Interestingly, more insoluble α‐syn aggregates formed in cells overexpressing SYNGR3 (Figures 2a and 2b). The SYNGR3 increased the levels of pS129 induced by PFFs (Figure 2c and S2a). Gradient lysis experiments revealed pS129 in the insoluble components of α‐syn‐HEK293 cells exposed to α‐syn‐HEK293 PFFs. The levels of insoluble pS129 were elevated in cells overexpressing SYNGR3 (Figure 2d and S2e).

**FIGURE 3:**
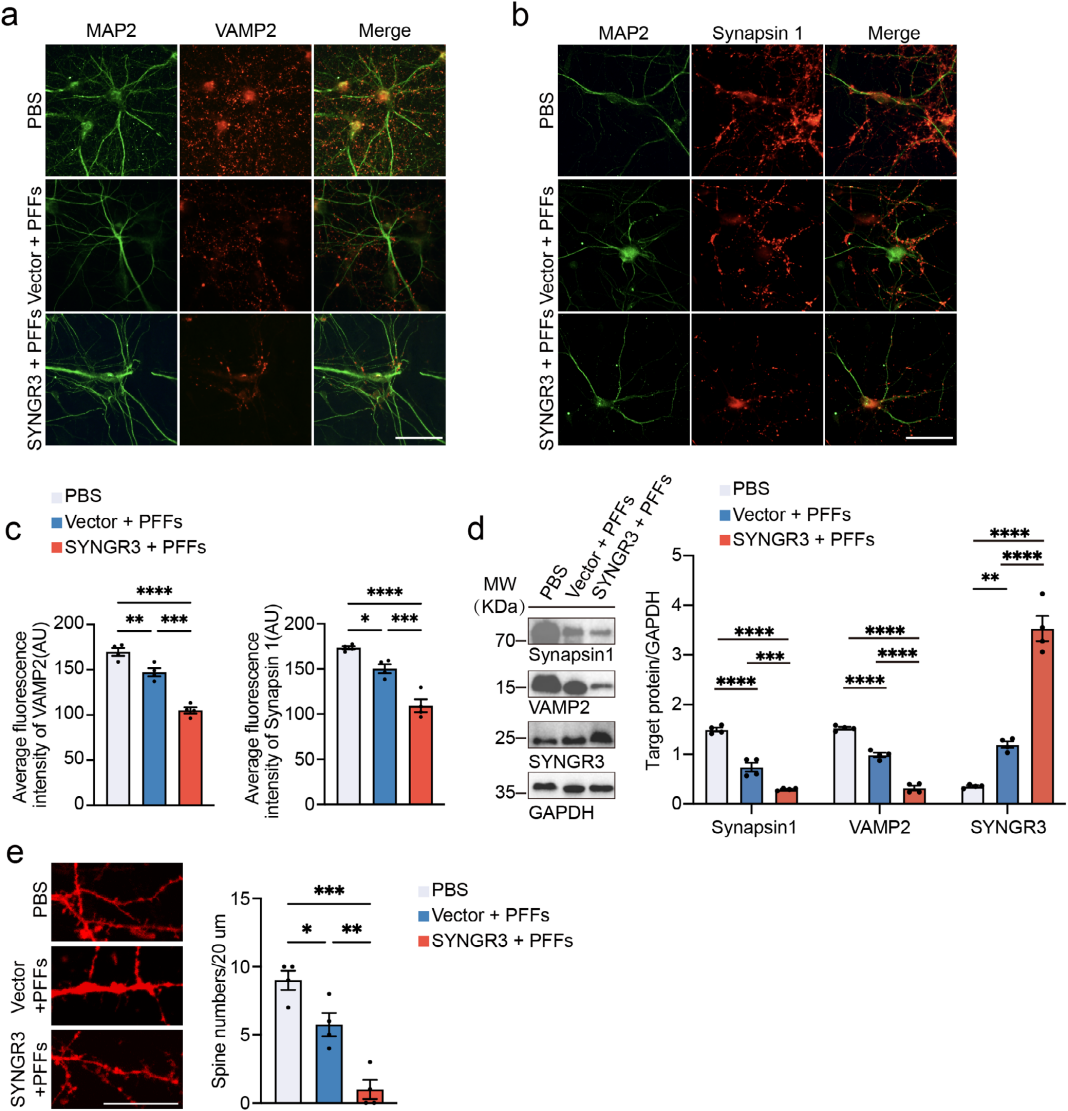
SYNGR3 enhances the synaptic degeneration induced by α‐syn PFFs in primary neurons. a. Primary neurons were infected with AAV‐vector or AAV‐SYNGR3 and then exposed to α‐syn PFFs. Shown are double‐labeling immunofluorescence of MAP2 (green) and VAMP2 (red). Scale bar = 50 μm. b. Double‐labeling immunofluorescence of MAP2 (green) and Synapsin 1 (red). Scale bar = 50 μm. c. Quantification of VAMP2 (left) and synapsin 1 (right) fluorescence intensity (*n* = 4 independent experiments). d. Western blot analysis of synapsin 1, VAMP2, and SYNGR3 levels in primary neurons infected with AAV‐GFP‐vector or AAV‐SYNGR3 and then treated with α‐syn PFFs. *Right*: Quantification of synapsin 1, VAMP2, and SYNGR3 levels (*n* = 4 independent experiments). e. Representative DiI‐stained images showing decreased spine density in primary neurons. The scale bar shows the quantification of the density of the spines (*n* = 4 independent experiments). Scale bar = 20 μm. Data are presented as mean ± SEM. *P* values were determined by one‐way ANOVA followed by Tukey's multiple comparison test. *p < 0.05, **p < 0.01, ***p < 0.001, ****p < 0.0001.

To determine whether SYNGR3 regulates α‐syn aggregation in primary neurons, the neurons were infected with AAV‐SYNGR3 or AAV‐vector for 4 days and then exposed to α‐syn PFFs for 7 days. Further, the immunofluorescence, gradient lysis, and Western blot analysis confirmed that the overexpression of SYNGR3 in primary neurons enhanced α‐syn aggregation induced by α‐syn PFFs (Figure 2e–g, S2b, and S2c). To determine whether SYNGR3 regulates the transcription of the SNCA gene, the RT‐PCR analysis was performed and found that the mRNA levels of α‐syn were not altered (Figure S2d and S2f). Furthermore, immunofluorescence revealed that the levels of synaptic proteins, including synapsin 1 and vesicle‐associated membrane protein 2 (VAMP2), were decreased in the neurons treated with AAV‐SYNGR3 (Figure 3a–c). These results were confirmed using the Western blot analysis (Figure 3d). Further, the density of dendritic spines was measured using the DiI staining. The results revealed that the overexpression of SYNGR3 led to a more serious reduction in the density of dendritic spines caused by α‐syn PFFs (Figure 3e). Together, these results indicate that SYNGR3 aggravates α‐syn aggregation and synaptic degeneration.

### 3.4 SYNGR3 Promotes Mitochondrial Dysfunction and Apoptosis

**FIGURE 4:**
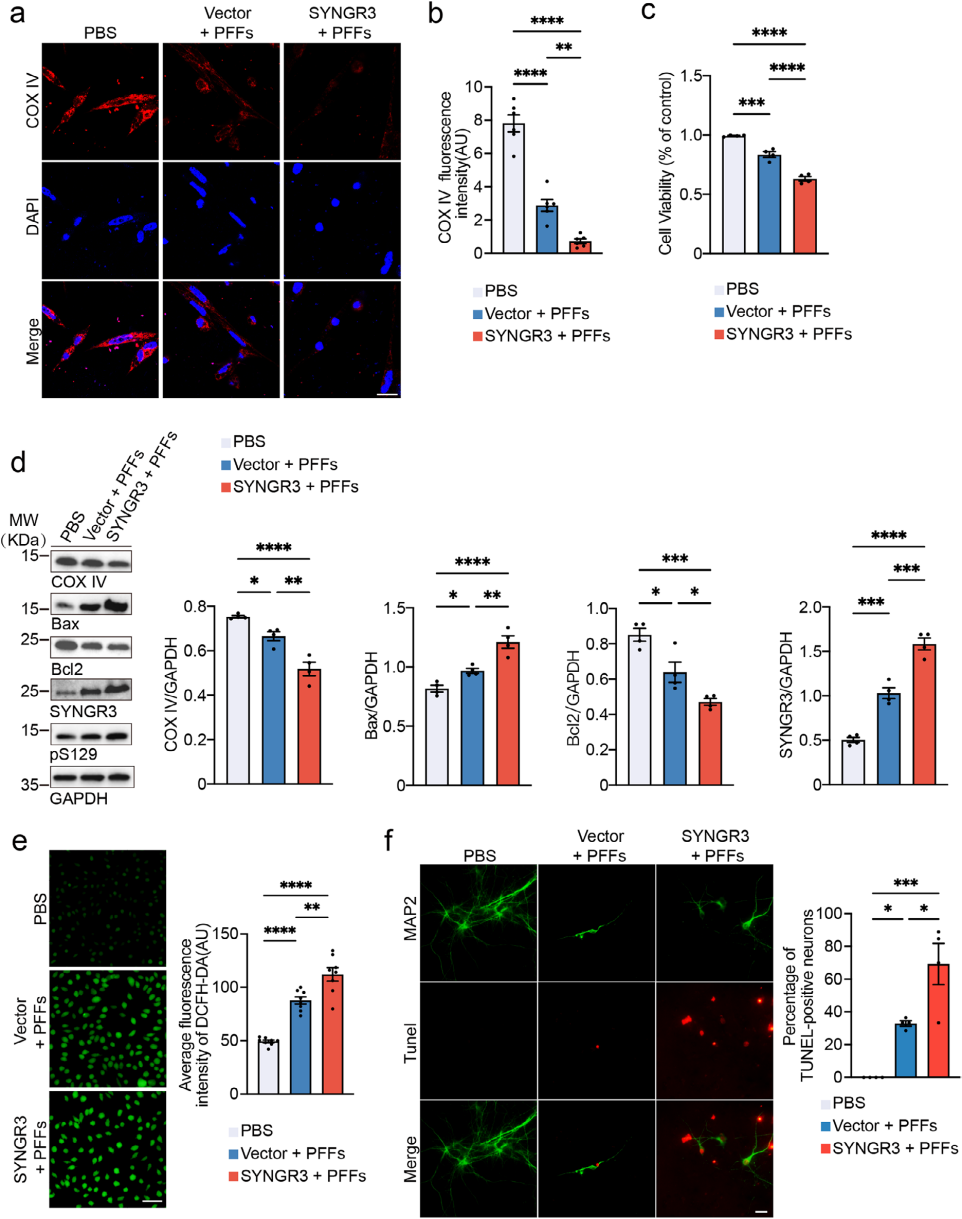
SYNGR3 promotes mitochondrial dysfunction, oxidative stress, and apoptosis. a. SH‐SY5Y cells were transfected with His‐SYNGR3 plasmids or His‐vector plasmids and then treated with α‐syn PFFs. Representative immunostaining images of COX IV (red). Scale bar = 20 μm. b. Quantification of COX IV fluorescence intensity (*n* = 6 independent experiments). c. CCK‐8 assay showing the viability of SH‐SY5Y cells (*n* = 4 independent experiments). d. Western blot analysis of COX IV, Bax, and Bcl2 in SH‐SY5Y cells transfected with His‐SYNGR3 plasmids or His‐vector plasmids and then treated with α‐syn PFFs. The bar graph shows the quantification of COX IV, Bax, Bcl2, and SYNGR3 (*n* = 4 independent experiments). e. ROS were detected using DCFH‐DA probe. Scale bar = 50 μm (*n* = 8 independent experiments). f. Double‐labeling immunofluorescence of MAP2 (green) and TUNEL (red) in primary neurons. The scale bar shows the quantification of the percentage of TUNEL‐positive neurons (*n* = 4 independent experiments). Scale bar = 20 μm. Data are presented as mean ± SEM. *P* values were determined by one‐way ANOVA followed by Tukey's multiple comparison test. *p < 0.05, **p < 0.01, ***p < 0.001, ****p < 0.0001.

Mitochondrial dysfunction contributes to neurodegeneration in PD [24]. Aggregation of α‐syn can directly damage mitochondrial function, reduce the activity of the respiratory chain complex, and generate a large amount of reactive oxygen species (ROS), which can further induce cell apoptosis. To test the effects of SYNGR3 on mitochondrial dysfunction induced by α‐syn PFFs, SH‐SY5Y cells were used as these cells are of neuronal origin, have a clear response to oxidative stress damage and have established use in PD mitochondrial studies [25, 26]. SH‐SY5Y cells were transfected with His‐tagged SYNGR3 plasmids and then treated with α‐syn PFFs. After 48 h, immunofluorescence and immunoblotting showed that cells exposed to PFFs presented decreased levels of the mitochondrial marker COX IV, and this reduction was further enhanced by SYNGR3 overexpression (Figure 4a b). Furthermore, the expression of the apoptotic protein Bax was increased, whereas the expression of antiapoptotic protein Bcl2 was reduced in the cells overexpressing SYNGR3 (Figure 4d). The DCFH‐DA staining revealed that the ROS levels were greater in cells overexpressing SYNGR3 (Figure 4e). The CCK8 assay revealed that SYNGR3 worsened PFF‐induced cytotoxicity (Figure 4c). Consistently, α‐syn PFFs induced apoptosis in primary neurons, which was exacerbated by SYNGR3 overexpression (Figure 4f). These results indicate that SYNGR3 enhanced the detrimental effect of α‐syn PFFs.

### 3.5 SYNGR3 Levels Are Correlated With α‐Syn Pathology in Vivo

**FIGURE 5:**
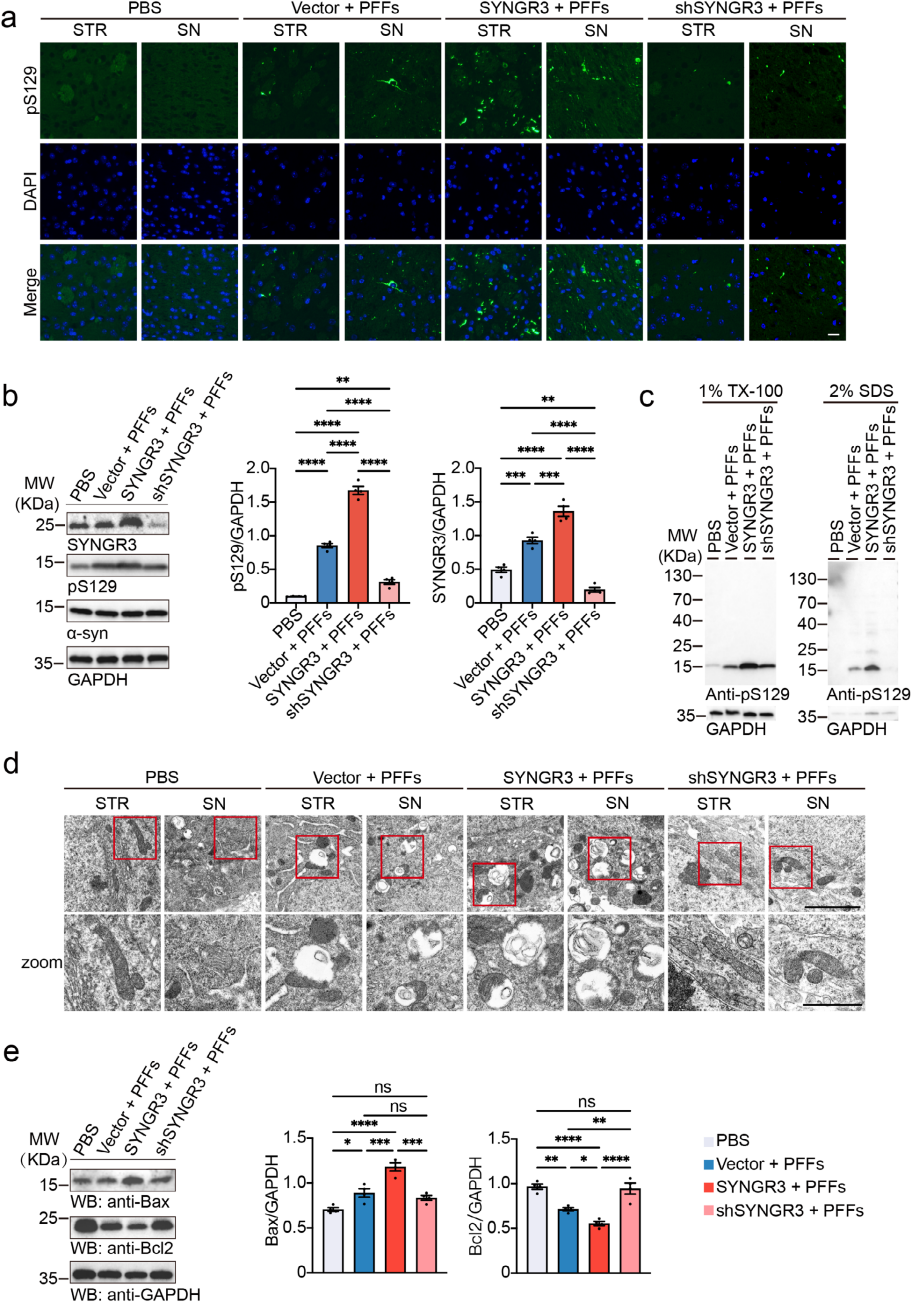
SYNGR3 promotes α‐syn pathology and mitochondrial dysfunction in TgA53T mice. a. Representative immunostaining images of pS129 (green) in the striatum (STR) and substantia nigra (SN) of mice 3 months after injection with α‐syn PFFs together with AAV‐SYNGR3, AAV‐shSYNGR3, or AAV‐Vector. Scale bar = 20 μm. b. Western blots of pS129 in striatal lysates of mice injected with α‐syn PFFs together with AAV‐SYNGR3, AAV‐shSYNGR3, or AAV‐Vector. The bar graph shows the quantification of pS129 and SYNGR3 (*n* = 4 mice per group). c. Sequential extraction of the ipsilateral striatum. d. Representative images of mitochondria in the ipsilateral striatum (STR) and substantia nigra (SN) of mice injected with α‐syn PFFs together with AAV‐SYNGR3, AAV‐shSYNGR3, or AAV‐Vector. *Top*: Scale bar = 2 μm. *Below*: Scale bar = 1 μm. e. Western blot analysis and quantification of Bax and Bcl2 in the ipsilateral striatum (*n* = 4 mice per group). Data are presented as mean ± SEM. *P* values were determined by one‐way ANOVA followed by Tukey's multiple comparison test. ns, Not significant; *p < 0.05, **p < 0.01, ***p < 0.001, ****p < 0.0001.

To further determine the effect of SYNGR3 on α‐syn pathology in vivo, α‐syn PFFs was injected along with AAV‐SYNGR3 that overexpresses SYNGR3, AAV‐shSYNGR3 that knocks down the expression of SYNGR3, or control AAV‐vector into the right striatum of 10‐week‐old TgA53T mice. One month after injection, phosphorylated α‐syn was detected in the striatum and substantia nigra in mice injected with α‐syn PFFs. α‐Syn deposition was exacerbated in the mice injected with AAV‐SYNGR3 and alleviated in the mice injected with AAV‐shSYNGR3 (Figure S3a and S3b). Three months later, more α‐syn deposition was detected in the brain in PFFs‐injected mice. Consistently with results observed at 1 month after treatment, over expression of SYNGR3 exacerbated while knockdown of SYNGR3 alleviated α‐syn pathology (Figure 5a and S3c). These results were confirmed by the Western blot analysis of pS129 in the striatal lysates (Figure 5b). Subsequent protein extraction experiments revealed that injection of AAV‐SYNGR3 increased pS129 accumulation in the insoluble fraction of the striatum, which was attenuated in mice injected with AAV‐shSYNGR3 (Figure 5c and S3d). These results indicate that the level of SYNGR3 was associated with the extent of α‐syn pathology.

### 3.6 SYNGR3 Promotes Mitochondrial Dysfunction in Vivo

To further verify the effects of SYNGR3 on mitochondrial function and synapses in the mouse model, tests using the electron microscopy and Western blotting were conducted using brain tissues collected from AAV‐injected mice. The mitochondrial morphology in the striatum and substantia nigra of the mice injected with α‐syn PFFs was disrupted, with damaged cristae and surrounding membranes. SYNGR3 overexpression intensified these abnormalities, while SYNGR3 knockdown mitigated them (Figure 5d). Injection of PFFs increased Bax and reduced Bcl2 levels in the striatum. This effect was enhanced by the overexpression of SYNGR3 and decreased by the knockdown of SYNGR3 (Figure 5e). Thus, SYNGR3 exacerbates the toxic effects induced by α‐syn PFFs in vivo.

### 3.7 SYNGR3 Exacerbates Neurotoxicity and Behavioral Impairments in TgA53T Mice

**FIGURE 6:**
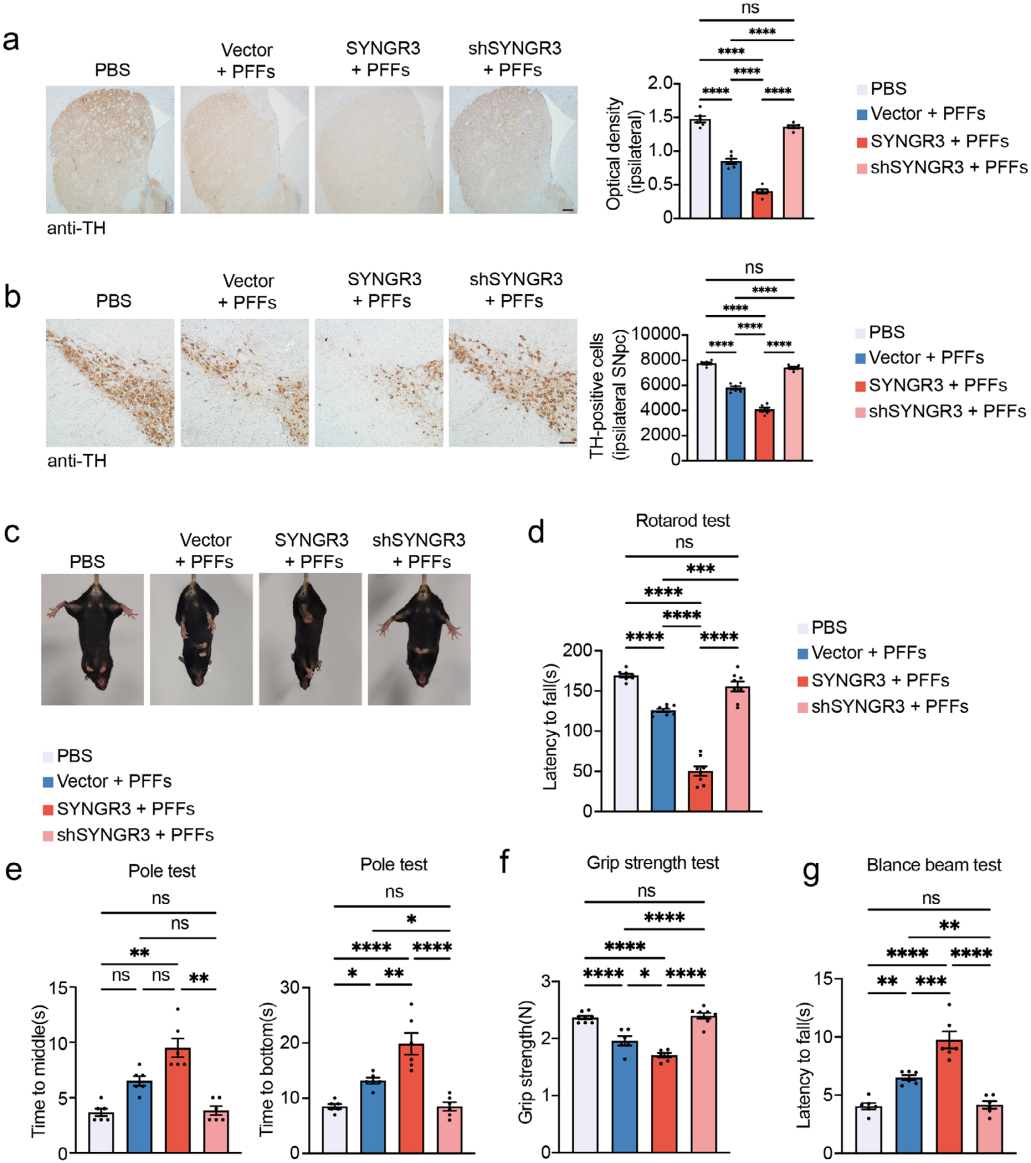
SYNGR3 exacerbates dopaminergic neurodegeneration and behavioral impairments in TgA53T mice. a. Representative images and quantification of TH‐positive terminals of the ipsilateral striatum of mice co‐injected with α‐syn PFFs and AAV‐SYNGR3, AAV‐shSYNGR3, or AAV‐Vector. Scale bar = 200 μm. b. Representative images of TH immunostaining of the SNpc of mice. Scale bar = 100 μm. *Right*: Quantification of TH‐positive cells in the substantia nigra pars compact (SNpc) (*n* = 6 mice per group). c. Representative images of the tail suspension test (*n* = 6 mice per group). (d‐g) Results of the rotarod test, pole test, grip strength test, and balance beam test (*n* = 6–8 mice per group). Data are presented as mean ± SEM followed by Tukey's multiple comparison test. *P* values were determined by one‐way ANOVA. ns, Not significant; *p < 0.05, **p < 0.01, ***p < 0.001, ****p < 0.0001.

Given that SYNGR3 accelerates α‐syn phosphorylation and mitochondrial dysfunction, we further explored whether its overexpression promotes degeneration of the nigrostriatal dopaminergic pathway. Compared with those in the control group, the number of TH‐positive neurons in the SNpc and the density of TH‐positive terminals in the striatum were lower in the SYNGR3‐overexpressing group (Figures 6a and 6b). The PD‐like motor impairments in mice were tested using the behavioral tests, including the tail suspension test, pole test, rotarod test, grip strength test, and balance beam test. In the tail suspension test, the PFF‐treated mice clumped their legs, which was more severe in the mice overexpressing SYNGR3 (Figure 6c). The rotarod test revealed shorter latency to fall in SYNGR3‐overexpression mice (Figure 6d). Motor performance in the pole test was reduced (Figure 6e), forelimb grip strength was weaker (Figure 6f), and the traversal time on the balance beam was longer (Figure 6g). These impairments were ameliorated by SYNGR3 knockdown. Thus, SYNGR3 aggravates dopaminergic neurodegeneration and PD‐like motor impairments in a mouse model of PD.

## 4 Discussion

The aggregation of α‐syn is key to the pathogenesis of PD. Under physiological conditions, α‐syn is enriched in presynaptic terminals and binds to synaptic membranes, where it participates in neurotransmitter release under physiological conditions [27, 28]. However, abnormal accumulation of α‐syn in neurons leads to synaptic dysfunction and neurotoxicity [13, 29, 30]. The factors that initiate α‐syn aggregation remain largely unknown.

This research has revealed the unique role that SYNGR3 may play in regulating α‐syn pathology. It should be noted that some other factors may also promote α‐syn pathology. Tau interacts with the C‐terminal of α‐syn through its microtubule‐binding repeat domain, promoting the formation of insoluble aggregated complexes [10, 31, 32]. Cofilin‐1 forms rod‐shaped complexes with α‐syn under oxidative stress and deposits on mitochondria, directly driving synaptic damage [33, 34, 35]. Additionally, it has been reported that the regulation of the O‐GlcNAc glycosylation cycle profoundly affects the aggregation tendency, degradation, and neuroinflammation triggered by α‐syn [36]. Different from the above studies, SYNGR3 is mainly located on the synaptic vesicle membrane and is part of a complex synaptic vesicle protein network [15]. This research showed that after exposure to α‐syn PFFs, the expression levels of synapsin1 and VAMP2 associated with SNARE complex [13, 28, 29] were decreased more dramatically when SYNGR3 was overexpressed (Figure 3a–3d). The functional imbalance of SYNGR3 may improperly bind a large amount of α‐syn to the vesicle membrane, possibly hindering its normal physiological functions related to SNARE complex assembly, leading to abnormal synaptic vesicle transport [37].

On the other hand, SYNGR3, as one of the members of the multigene family of integral synaptic vesicle proteins, differs from SYNGR2 and SYNGR4, is mainly expressed in neurons, especially in brain regions rich in dopaminergic neurons [15, 16, 38, 39, 40]. There is currently no clear evidence indicating that the other members are directly associated with PD or α‐syn pathology. This regional specificity and uniqueness make SYNGR3 particularly important for the synaptic homeostasis of specific types of neurons. We speculate that under PD‐related stress, the expression or stability of SYNGR3 may be more severely impaired and become a breakthrough point for PD‐related pathology. In contrast, the other family members may perform more basic vesicle functions or play roles in other physiological or pathological conditions.

As previously reported, the initiation of α‐syn begins approximately decades before the appearance of typical movement symptoms in PD [41, 42]. This study mainly focused on how SYNGR3 affects the α‐syn pathology in the early stage and tries to seek potential targets for early intervention. The results showed that SYNGR3 binds to α‐syn and promotes its aggregation, gain of function and loss of function experiments in vivo were conducted and confirmed the role of this target in the disease. One of the limitations of this study is that the effects of SYNGR3 overexpression/knockdown in the cellular models were not thoroughly tested. Although the results suggest that disrupting the interaction between SYNGR3 and α‐syn may alleviate α‐syn pathology, more experiments are needed to develop small molecules or peptides that can do so.

## 5 Conclusion

Overall, this study discovered that SYNGR3 interacts with α‐syn and promotes its aggregation, inducing PD‐related pathologies, including synaptic dysfunction, mitochondrial dysfunction, oxidative stress, and neuronal apoptosis. Thus, SYNGR3 may serve as a potential therapeutic target to alleviate the progression of PD.

## Author Contributions

Z.Z. and J.Y. conceived the project and designed the experiments. X.W. and J.Y. performed most of the experiments. Z.W., Z.L., and W.T. helped with the in vitro experiments and data analysis. X.W. and W.T. wrote the manuscript with input from all the authors.

## Funding

This study was supported by the National Natural Science Foundation of China, 82201395, 82271447.

## Ethics Statement

The experimental procedure was approved by the Institutional Animal Care and Use Committee (IACUC) of Renmin Hospital of Wuhan University. The IACUC approval number is 20230806.

## Conflicts of Interest

The authors declare no conflicts of interest.

## Supporting information


**Figure S1:** SYNGR3 increase in TgA53T mice, interacts with α‐syn and promotes its aggregation. Related to Figure 1.
Quantification of SYNGR3 and pS129 levels (*n* = 6 mice per group).
Representative Western blot and quantification of SYNGR3 and pS129 in the striatum of age‐matched WT and TgA53T mice at different ages (*n* = 4 mice per group).
Kinetics of α‐syn (1 mg/mL) fibrillization in the presence of SYNGR3 at different concentration in a real‐time Th T fluorescence assay. Data are presented as mean ± SEM. *P* values were determined by *t*‐test or one‐way ANOVA followed by Tukey's multiple comparison test. ns, Not significant; *p < 0.05, **p < 0.01, ***p < 0.001, ****p < 0.0001.
**Figure S2:** SYNGR3 promotes α‐syn phosphorylation and aggregation rather than influences the translation of α‐syn. Related to Figure 2.
Quantification of SYNGR3 in Figure 2c. (*n* = 4 independent experiments).
Quantification of SYNGR3 in Figure 2f. (*n* = 4 independent experiments).
Primary neurons were infected with AAV‐GFP‐vector or AAV‐GFP‐SYNGR3 and then treated with α‐syn PFFs. The bar graph shows the quantification of α‐syn in 1% TX‐100 and 2% SDS fractions, respectively (*n* = 4 independent experiments). The phosphate‐buffered saline (PBS) group was used for normalization.
Relative mRNA level of α‐syn in primary neurons infected with AAV‐vector or AAV‐SYNGR3 and then treated with α‐syn PFFs (*n* = 4 independent experiments).
α‐Syn‐HEK293 cells were transfected with His‐SYNGR3 plasmids or control plasmids and then treated with α‐syn PFFs. The cells were sequentially extracted with 1% TX‐100 and 2% SDS. The bar graph shows the quantification of α‐syn and pS129 in 1% TX‐100 and 2% SDS fractions, respectively (*n* = 4 independent experiments).
Relative mRNA level of α‐syn in α‐syn‐HEK293 cells initially transfected with His‐SYNGR3 plasmids or control plasmids and then treated with α‐syn PFFs (*n* = 4 independent experiments). The PBS group was used for normalization. Data are presented as mean ± SEM. *P* values were determined by one‐way ANOVA followed by Tukey's multiple comparison test. ns, Not significant; *p < 0.05, **p < 0.01, ***p < 0.001, ****p < 0.0001.
**Figure S3:** SYNGR3 promotes α‐syn pathology in vivo. Related to Figure 5.
Representative immunostaining images of pS129 (green) in the striatum (STR) and substantia nigra (SN) of TgA53T mice injected with α‐syn PFFs together with AAV‐SYNGR3, AAV‐shSYNGR3, or AAV‐Vector at 1 month post‐injection. Scale bar = 20 μm.
Quantification of pS129 fluorescence intensity in STR and SN of mice at 1 month post‐injection (*n* = 4 mice per group).
Quantification of pS129 fluorescence intensity in STR and SN of mice at 3 months post‐injection (*n* = 4 mice per group).
The bar graph shows the quantification of pS129 in 1% TX‐100 and 2% SDS fractions in Figure 5c (*n* = 4 mice per group). Data are presented as mean ± SEM. *P* values were determined by one‐way ANOVA followed by Tukey's multiple comparison test. ns, Not significant; *p < 0.05, **p < 0.01, ***p < 0.001, ****p < 0.0001.

**Table S1.** Primers sequences used for the RT‐PCR analysis.

## Data Availability

The data that support the findings of this study are available from the corresponding author upon reasonable request.
