# Supplementary material for: SYNGR3 Accelerates α‐Synuclein Aggregation and Neurodegeneration in Parkinson's Disease
Source: CNS Neurosci Ther. 2026 Mar 24;32(3):e70842. doi: 10.1002/cns.70842 (PMC13093712; doi:10.1002/cns.70842)
Supplement: Supplementary file 1 — Figure S1: SYNGR3 increase in TgA53T mice, interacts with α‐syn and promotes its aggregation. Related to Figure 1. Quantification of SYNGR3 and pS129 levels (n = 6 mice per group). Representative Western blot and quantification of SYNGR3 and pS129 in the striatum of age‐matched WT and TgA53T mice at different ages (n = 4 mice per group). Kinetics of α‐syn (1 mg/mL) fibrillization in the presence of SYNGR3 at different concentration in a real‐time Th T fluorescence assay. Data are presented as mean ± SEM. P values were determined by t‐test or one‐way ANOVA followed by Tukey's multiple comparison test. ns, Not significant; *p < 0.05, **p < 0.01, ***p < 0.001, ****p < 0.0001. Figure S2: SYNGR3 promotes α‐syn phosphorylation and aggregation rather than influences the translation of α‐syn. Related to Figure 2. Quantification of SYNGR3 in Figure 2c. (n = 4 independent experiments). Quantification of SYNGR3 in Figure 2f. (n = 4 independent experiments). Primary neurons were infected with AAV‐GFP‐vector or AAV‐GFP‐SYNGR3 and then treated with α‐syn PFFs. The bar graph shows the quantification of α‐syn in 1% TX‐100 and 2% SDS fractions, respectively (n = 4 independent experiments). The phosphate‐buffered saline (PBS) group was used for normalization. Relative mRNA level of α‐syn in primary neurons infected with AAV‐vector or AAV‐SYNGR3 and then treated with α‐syn PFFs (n = 4 independent experiments). α‐Syn‐HEK293 cells were transfected with His‐SYNGR3 plasmids or control plasmids and then treated with α‐syn PFFs. The cells were sequentially extracted with 1% TX‐100 and 2% SDS. The bar graph shows the quantification of α‐syn and pS129 in 1% TX‐100 and 2% SDS fractions, respectively (n = 4 independent experiments). Relative mRNA level of α‐syn in α‐syn‐HEK293 cells initially transfected with His‐SYNGR3 plasmids or control plasmids and then treated with α‐syn PFFs (n = 4 independent experiments). The PBS group was used for normalization. Data are presented as mean ± SEM. P valu [file CNS-32-e70842-s001.docx]

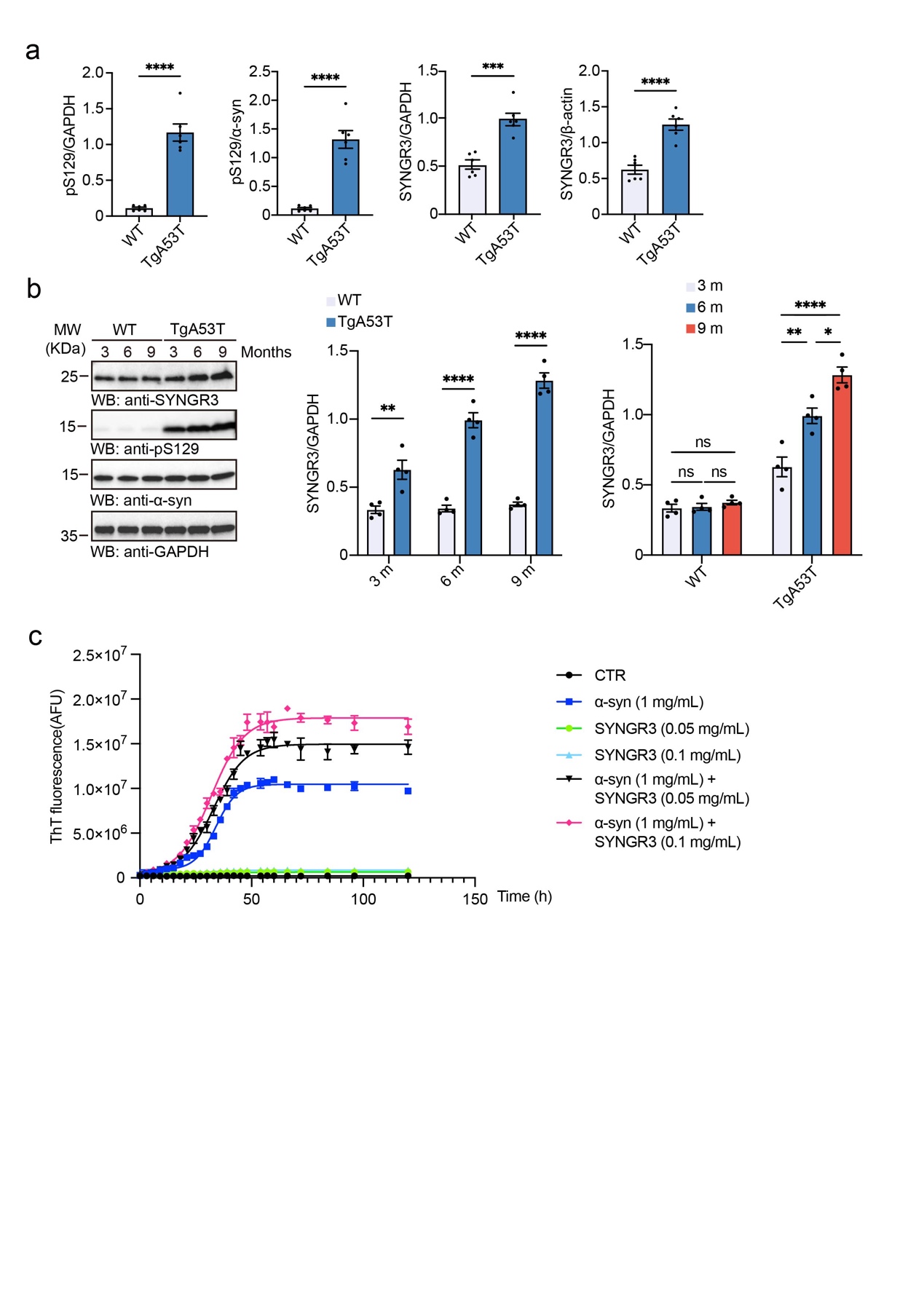


**Fig. S1**. **SYNGR3 increased in TgA53T mice, interacts with α-syn and promotes its aggregation. Related to Fig. 1.**

1. Quantification of SYNGR3 and pS129 levels (n = 6 mice per group).
2. Representative Western blot and quantification of SYNGR3 and pS129 in the striatum of age-matched WT and TgA53T mice at different ages (n = 4 mice per group).
3. Kinetics of α-syn (1 mg/ml) fibrillization in the presence of SYNGR3 at different concentration in a real-time Th T fluorescence assay. Data are presented as mean ± SEM. *P* values were determined by *t*-test or one-way ANOVA followed by Tukey’s multiple comparison test. ns, not significant; **P* < 0.05, ***P* < 0.01, ****P* < 0.001, *****P* < 0.0001.


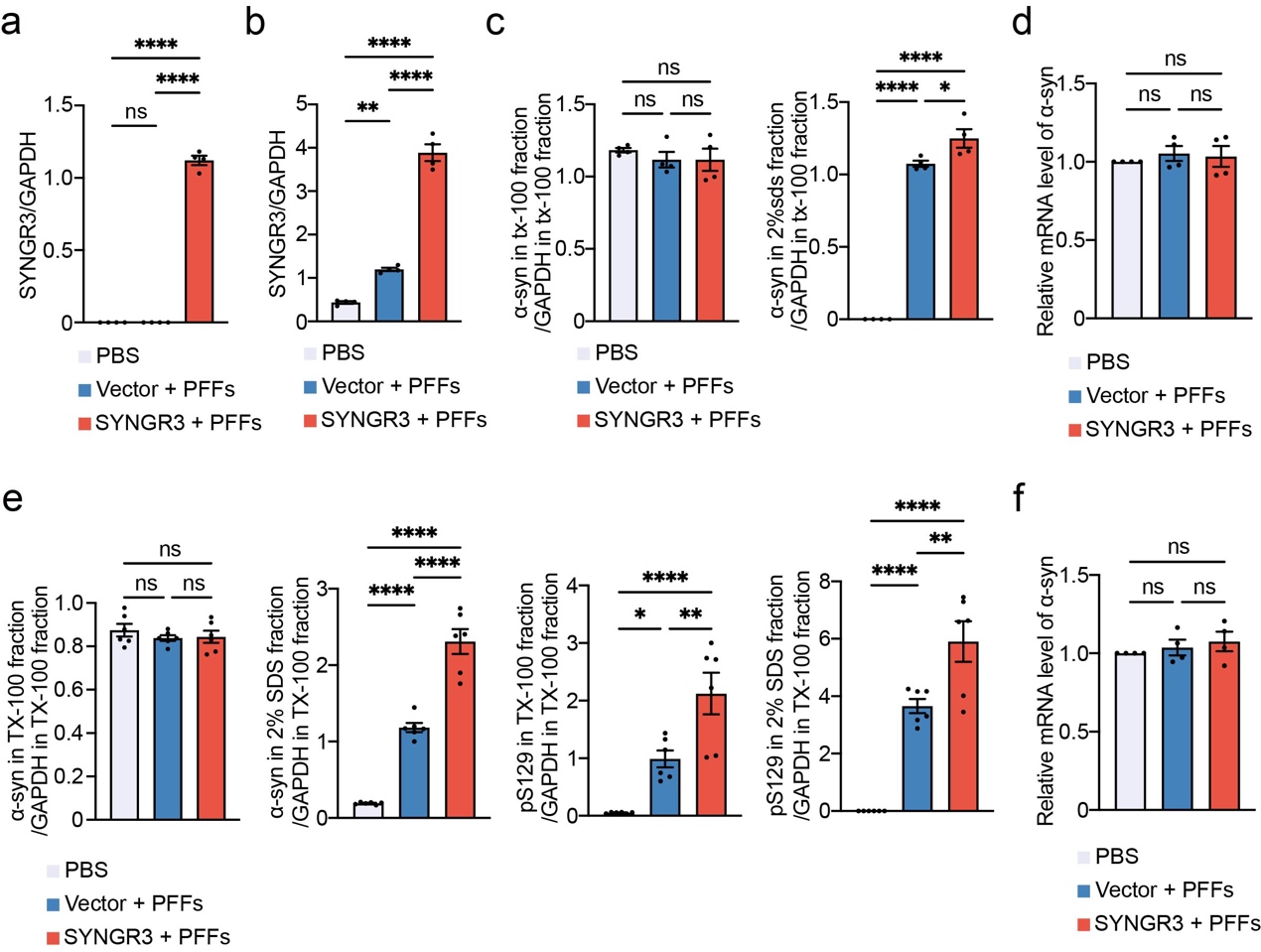


**Fig. S2. SYNGR3 promotes α-syn phosphorylation and aggregation rather than influences the translation of α-syn. Related to Fig. 2.**

1. Quantification of SYNGR3 in fig 2c. (n = 4 independent experiments).
2. Quantification of SYNGR3 in fig 2f. (n = 4 independent experiments).
3. Primary neurons were infected with AAV-GFP-vector or AAV-GFP-SYNGR3 and then treated with α-syn PFFs. The bar graph shows the quantification of α-syn in 1% TX-100 and 2% SDS fraction respectively (n = 4 independent experiments). PBS group was performed normalization.
4. Relative mRNA level of α-syn in primary neurons infected with AAV-vector or AAV-SYNGR3 and then treated with α-syn PFFs (n = 4 independent experiments).
5. α-Syn-HEK293 cells were transfected with His-SYNGR3 plasmids or control plasmids and then treated with α-syn PFFs. The cells were sequentially extracted with 1% TX-100 and 2% SDS. The bar graph shows the quantification of α-syn and pS129 in 1% TX-100 and 2% SDS fraction respectively (n = 4 independent experiments).
6. Relative mRNA level of α-syn in α-syn-HEK293 cells transfected with His-SYNGR3 plasmids or control plasmids and then treated with α-syn PFFs (n = 4 independent experiments). PBS group was performed normalization. Data are presented as mean ± SEM. *P* values were determined by one-way ANOVA followed by Tukey’s multiple comparison test. ns, not significant; **P* < 0.05, ***P* < 0.01, ****P* < 0.001, *****P* < 0.0001.


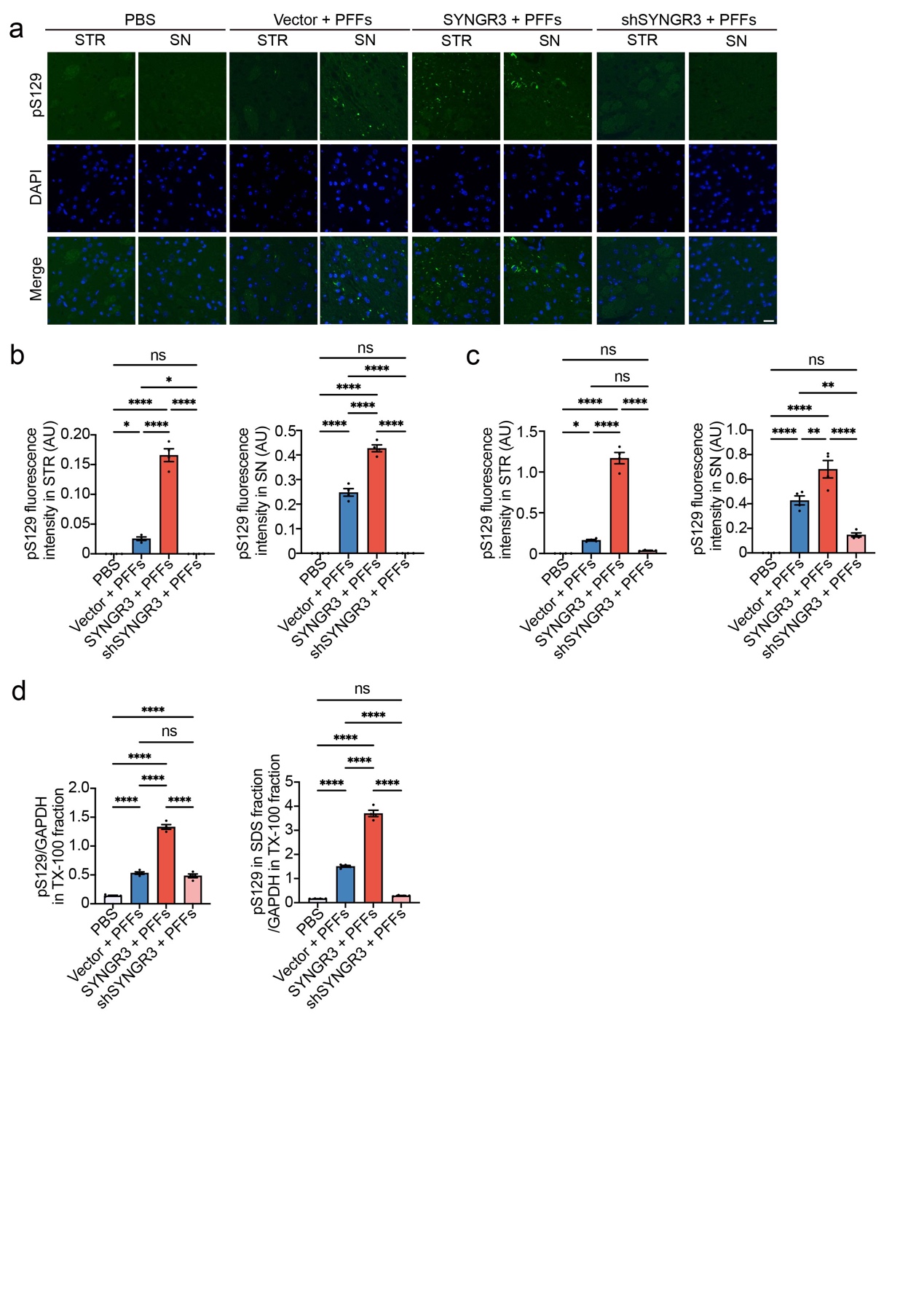


**Fig. S3. SYNGR3 promotes α-syn pathology *in vivo*. Related to Fig. 5.**

1. Representative immunostaining images of pS129 (green) in the striatum (STR) and substantia nigra (SN) of TgA53T mice injected with α-syn PFFs together with AAV-SYNGR3, AAV-shSYNGR3, or AAV-Vector at 1 month post-injection. Scale bar = 20 μm.
2. Quantification of pS129 fluorescence intensity in STR and SN of mice at 1 month post-injection (n = 4 mice per group).
3. Quantification of pS129 fluorescence intensity in STR and SN of mice at 3 months post-injection (n = 4 mice per group).
4. The bar graph shows the quantification of pS129 in 1% TX-100 and 2% SDS fraction in fig 5c (n = 4 mice per group). Data are presented as mean ± SEM. *P* values were determined by one-way ANOVA followed by Tukey’s multiple comparison test. ns, not significant; **P* < 0.05, ***P* < 0.01, ****P* < 0.001, *****P* < 0.0001.
